# The exposure to volatile organic chemicals associates positively with rheumatoid arthritis: a cross-sectional study from the NHANES program

**DOI:** 10.3389/fimmu.2023.1098683

**Published:** 2023-06-19

**Authors:** Ting Lei, Hu Qian, Junxiao Yang, Yihe Hu

**Affiliations:** ^1^Department of Orthopedic Surgery, Hunan Engineering Research Center of Biomedical Metal and Ceramic Implants, National Clinical Research Center of Geriatric Disorder, Xiangya Hospital, Central South University, Changsha, China; ^2^Department of Orthopedic Surgery, Affiliated Hospital of Zunyi Medical University, Zunyi, China; ^3^Department of Orthopedic Surgery, The First Affiliated Hospital, College of Medicine, Zhejiang University, Hangzhou, China

**Keywords:** volatile organic chemicals, rheumatoid arthritis, public health, environment pollution, national health and nutrition examination survey

## Abstract

**Introduction:**

Rheumatoid arthritis (RA) is an autoimmune disease and closely associated with both genetic and environmental factors. Volatile organic chemicals (VOC), a common environment pollutant, was associated with some autoimmune diseases, while whether VOC exposure or which VOC leads to RA is yet clarified.

**Methods:**

A cross-sectional study using data from the 6 survey cycles (2005-2006, 2011-2012, 2013-2014, 2015-2016, 2017-2018, 2017-2020) of NHANES program was performed. The RA or non-arthritis status of participant was identified through a questionnaire survey. The quantile logistic regression method was used for correlation analysis between VOC metabolites (VOCs) in urine and RA. The covariates included age, gender, race, educational level, marital status, total energy intake, physical activity, smoking, hypertension, diabetes, urine creatinine, albumin and marihuana use.

**Results:**

A total of 9536 participants (aged 20 to 85) with 15 VOCs, comprising 618 RA and 8918 non-arthritis participants, was finally included for analysis. Participants in the RA group showed higher VOCs in urine than that in the non-arthritis group. A positive association between 2 VOCs (AMCC: Q4: OR=2.173, 95%CI: 1.021, 4.627. 3HPMA: Q2: OR=2.286, 95%CI: 1.207 - 4.330; Q4: OR=2.663, 95%CI: 1.288 -5.508.) and RA was detected in the model 3, which was independent of all the covariates. The relative parent compounds of the two VOCs included N,N-Dimethylformamide and acrolein.

**Discussion:**

These findings suggested that the VOC exposure significantly associated with RA, providing newly epidemiological evidence for the establishment that environmental pollutants associated with RA. And also, more prospective studies and related experimental studies are needed to further validate the conclusions of this study.

## Introduction

Rheumatoid arthritis (RA), as one of the most prevalent chronic inflammatory diseases, is characterized with systemic autoimmune disorders and inflammatory synovitis, affecting 0.5–1.0% of the population over the world (1–3). Despite the ambiguity of the exact cause, accumulating evidence have been provided that the incidence and development of RA is a multifactorial interaction and closely associated with both genetic and environmental factors (4). According to previous studies, the genetic influences accounted for 50–60% of the risk for developing RA, while the remainder may be explained by environmental effect (4, 5). The association between environmental pollutants and RA is well established, such as smoking, while growing evidence suggests other environmental pollutants (6–8). For example, the exposure to pesticides, insecticide and traffic pollution was reported to be associated with RA (9–12). And also, the exposure to dust appeared to be a risk factor for RA (13).

The effect of environment pollutants on the risk of RA seems to have been answered in the affirmative, and even some studies have begun to explore the underlying molecular mechanism (6). Nevertheless, there were little specific pollutants strongly demonstrated to be associated with the risk of RA by epidemiological studies. For example, the exposure to polycyclic aromatic hydrocarbon (PAH) was found to be significantly associated with the occurrence of RA (14). And several Per-/polyfluoroalkyl substances (PFASs) were found to be significantly associated with the change of specific immune marker levels, suggesting that the exposure may lead to the increase of RA risk (15). These studies directly investigating the relationship between specific environmental pollutants and risk for RA provided us with more powerful evidence, which would help us implement more precise environmental protection and more effective prevention from RA. Therefore, it was imperative to identify specific pollutants leading to RA.

Volatile organic compounds (VOC) are common organic pollutants in the air, which could be produced by both human industry activity and natural sources (16). Compared with other pollutants exposed to people in specific environments, the widespread presence of VOC in the air results in easier and more common exposure to the general population, of which the exposure approach mainly comprises pulmonary inhalation, digestive absorption and cutaneous infiltration (17). The exposure to VOC has been found to be potentially associated with the risk of several autoimmune disorders through the abnormal activation or overactivation of immune cells (18, 19). These autoimmune diseases included pulmonary disease, atherosclerosis, and rheumatoid arthritis. Similarly, there were very little studies providing strong evidence that the risk of RA is actually associated with some specific pollutants, such as certain VOC.

Herein, we carried out this cross-sectional study aimed to investigate the association between RA and the exposure to specific volatile organic compounds metabolites (VOCs) in urine, which could specifically reflect the degree of exposure to a particular VOC parent. To our best knowledge, this is the first study to systematically investigate whether the exposure to specific VOC is associated with RA, which would provide new epidemiological evidence about the association of VOC exposure and RA, and help us develop more precise prevention and control measures against RA.

## Data and methods

### Data source

In this study, all the data was obtained from the National Health and Nutrition Examination Survey (NHANES) program (https://www.cdc.gov/nchs/nhanes). Performed very two years, the NHANES program is a series of population-based surveys designed to investigate the nutrition and health status of children, as well as adults in the USA. The participants included in this program was sampled using the multistage stratified sampling method combined with oversampling for certain subgroups, such as people older than 60 and people of other races in the USA. Various data including demographic data, examination data, laboratory data and questionnaire data were obtained from the participants. The investigation protocol of NHANES program was reviewed and approved by the National Center for Health Statistics (NCHS), a division of Centers for Disease Control and Prevention (CDC). Before inclusion, all the participants received an informed consent form setting out details of this program, and would sign their consent form. In this study, we used the data of the volatile organic compounds metabolites in urine to reflect the exposure to volatile organic compounds. A total of 6 survey cycles of the NHANES program (2005-2006, 2011-2012, 2013-2014, 2015-2016, 2017-2018, 2017-2020) was used and combined for association analysis between VOC exposure and RA.

### Measure of urine VOCs

The quantitative detection of VOCs in human urine was performed using ultra performance liquid chromatography coupled with electrospray tandem mass spectrometry (UPLC-ESI/MSMS) as described in previous studies (20). The Acquity UPLC^®^ HSS T3 (Part no. 186003540, 1.8 µm x 2.1 mm x 150 mm, Waters Inc.) column, with 15 mM ammonium acetate and acetonitrile as the mobile phases, was applied for chromatographic separation. In brief, the eluent from the column is firstly ionized using an electrospray interface to generate and transmit negative ions into the mass spectrometer. Then the comparison of relative response factors (ratio of native analyte to stable isotope labeled internal standard) with known standard concentrations yields individual analyte concentrations (https://wwwn.cdc.gov/nchs/data/nhanes/2015-2016/labmethods/UVOC_UVOCS_I_MET.pdf). More details of the laboratory method detecting urine VOCs could be found in the website (https://wwwn.cdc.gov/nchs/data/nhanes/2015-2016/labmethods/UVOC_UVOCS_I_MET.pdf).

### Assessment of RA condition

The RA or non-arthritis status of participant was identified through a questionnaire survey. In brief, participants will firstly be asked ‘Did a doctor or other healthcare professional ever tell you that you have arthritis?’. If the answer was “yes,” a follow-up question “What type of arthritis was it?” would be asked. According to the answers of the 2 questions, participants were grouped into the RA or non-RA subgroups. If the participant reported “no” for the first question, the participant was grouped into the non-arthritis group. If the participant reported “yes” for the first question and “RA” for the second question, the participant was grouped into the RA group.

### Assessment of covariates

The demographic data (age, gender, race, educational level, marital status), body mass index (BMI), dietary information (total energy intake), life behavior characteristics (moderate work activity, smoking status), concurrent disease (diabetes, hypertension), kidney function (urine creatinine, albumin) and marijuana use of participants were collected and set as the covariates for statistical analysis. All the participants included in this study were over 20 years old. The race information included 5 groups (Mexican American, other Hispanic, non-Hispanic White, non-Hispanic Black and other race). The education level was divided into 6 subgroups (less than 9th grade, 9-11th grade, high school graduate, some college or aa degree, college graduate or above, refused, don’t know), and the marital status was separated into 7 states (married, widowed, divorced, separated, never married, living with partner, refused). The BMI information of each participant was obtained from the examination data, which was calculated by dividing weight by the square of height (kg/m^2^). Then the participants could be further separated into 3 subgroups, including normal weight (BMI < 25), overweight (25≤BMI < 30), and obesity (BMI≥30) (21). The information of total energy intake could be obtained from the first 24-h recall questionnaire. The activity status of participants was identified into 2 status (yes or no) depending on whether they performed moderate activity work. The smoking status of participants were divided into non-users (smoking less than 100 cigarettes), past smoking (smoking more than 100 cigarettes, but no smoking now) and current smoking (smoking more than 100 cigarettes, now smoking) by smoking questionnaire. The relative information of smoking status could be found in the questionnaire data. The hypertension diagnosis was established when a systolic blood pressure > 140 mmHg, or a diastolic blood pressure > 90 mmHg was detected. The diabetes diagnosis was identified by the questionnaire data, or the detection of a fasting plasma glucose ≥ 7.0 mmol/L, or the use of antidiabetic drugs. The information of urine creatinine and albumin was acquired from the Laboratory test data of the NHANES program. The marijuana use information was acquired from the questionnaire data of NHANES program (22). According to the questionnaire data, the status of marijuana use was grouped into two types (ever or non-users).

### Statistical analysis

The correlation analysis between exposure to VOCs in urine and RA was performed in this cross-sectional study. Firstly, all the included participants were grouped into RA or non-arthritis subgroups according to their arthritis evaluation. Then the comparation analysis of baseline data between RA and non-arthritis subgroups was carried out by using the Chi-square test for categorical data and Kruskal-Wallis test for quantitative data respectively. Meanwhile, the concentration of various urine VOCs between RA and non-arthritis subgroups was compared by the Kruskal-Wallis test. And also, the concentration of various urine VOCs in the RA population with different smoking status was compared by the Kruskal-Wallis test. Next, 3 quantile logistic regression models with different covariates were constructed to analyze the association between the urine VOCs and RA. In the quantile logistic regression analysis, the concentration of all VOCs is divided into 4 concentration intervals according to the concentration from low to high, and the number of people in each concentration interval is roughly equal. The non-adjust model was firstly constructed to directly analyze the association between urine VOCs and RA, and the other 2 model were subsequently applied for further analysis. The model 1 was adjusted by age, gender, race, educational level, marital status, urinary creatinine and albumin. The model 2 was adjusted by age, gender, race, educational level, marital status, total energy intake, physical activity, smoking, hypertension, diabetes, urinary creatinine and albumin. The model 3 was adjusted by age, gender, race, educational level, marital status, total energy intake, physical activity, smoking, hypertension, diabetes, urinary creatinine, urinary albumin and marijuana use. In addition, the non-linear relationship between RA and 15 VOCs was analyzed using the smooth curve fitting method. Subgroup analysis was performed to analyze the association between VOCs and RA in population identified with both non-users smoking and non-users marijuana status. Due to the combination of multiple survey cycles, the baseline data and VOCs comparation results between RA and non-arthritis groups were adjusted by sampling weight. The sensitivity analysis was performed through restricting the association analysis in population without smoking and marijuana use history. In addition, multiple covariates were included in this study for quantile regression analysis. A *p* < 0.05 was identified as statistically significant in all the statistical analysis. The R software (https://www.r-project.org/), with the help of EmpowerStats (https://www.empowerstats.com), were used together for statistical analysis.

## Results

### Screening of eligible participants

**Figure 1:**
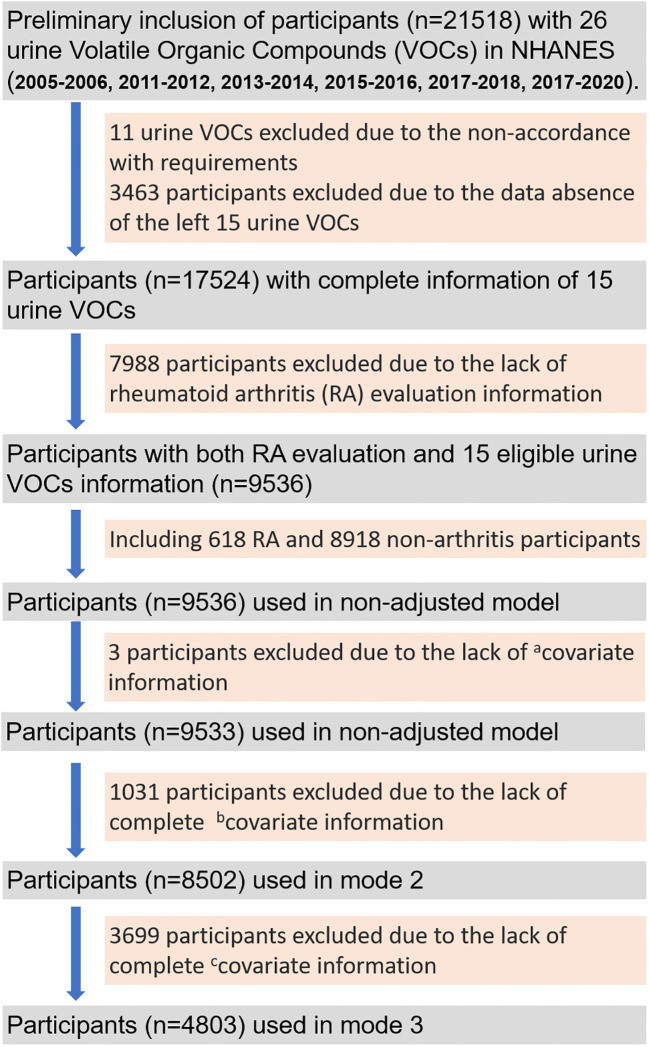
The flow gram of screening out eligible participants from 6 survey cycles (2005-2006, 2011-2012, 2013-2014, 2015-2016, 2017-2018, 2017-2020) of the National Health and Nutrition Examination Survey (NHANES) program. ^a^covariate information: age, gender, race, educational level, marital status, urine creatinine and albumin; ^b^covariate information: total energy intake, physical activity, smoking, hypertension, diabetes; ^c^covariate information: marihuana use.

As shown in **Figure 1**, there were 21518 participants preliminarily included with 26 urine VOCs information. After removing participants without complete information of 15 eligible VOCs, 17524 participants were left. And a total of 9536 participants with complete RA evaluation and covariates information was finally included for analysis using the non-adjusted model and model 1, comprising 618 RA participants and 8918 non-arthritis participants. The model 1 was adjusted with age, gender, race, educational level and marital status. A total of 8505 participants were finally included for analysis using the model 2, which was adjusted by age, gender, race, educational level, marital status, total energy intake, physical activity, smoking, hypertension and diabetes. And 4803 participants were finally included for analysis using the model 3, which was adjusted by age, gender, race, educational level, marital status, total energy intake, physical activity, smoking, hypertension, diabetes, urine creatinine, albumin and marihuana use.

### The baseline data comparation of included participants

The comparation results of baseline or covariates data between RA and non-arthritis subgroups were summarized in the **Table 1**. All the participants were grouped into RA and non-arthritis subgroups. A significant difference was detected between the 2 subgroups in terms of age, gender, race composition, education level composition, marital status, BMI, smoking, hypertension and diabetes, which was absent in terms of physical activity. Participants in the RA subgroup showed older age, higher percentage of women, higher BMI, higher percentage of overweight and obesity people than that in the non-arthritis subgroup. In addition, the percentage of smoking, hypertension and diabetes participants in the RA subgroup was significantly higher than that in the non-arthritis subgroup. In addition, the baseline data of RA and non-arthritis groups adjusted by sampling weight was summarized in **Table S1**, the analysis results were almost consistent with that adjusted without sampling weight.

**Table 1:** Baseline data of included participants.

characteristics	No arthritis (n=8918)	RA (n=618)	*p*
**Age (years, mean±SD)**	44.57 ± 16.61	60.65 ± 13.28	***
**Gender, n (%)**			***
Male	4732 (53.06%)	281 (45.47%)	
female	4186 (46.94%)	337 (54.53%)	
**Race hispanic origin, n (%)**			***
Mexican American	1349 (15.13%)	76 (12.30%)	
Other Hispanic	813 (9.12%)	49 (7.93%)	
Non-Hispanic White	3108 (34.85%)	234 (37.86%)	
Non-Hispanic Black	2199 (24.66%)	209 (33.82%)	
Other Race	1449 (16.25%)	50 (8.09%)	
**Education level, n (%)**			***
Less Than 9th Grade	777 (8.71%)	88 (14.24%)	
9-11th Grade	1198 (13.43%)	97 (15.70%)	
High School Graduate	2044 (22.92%)	151 (24.43%)	
Some College or AA degree	2721 (30.51%)	206 (33.33%)	
College Graduate or above	2169 (24.32%)	75 (12.14%)	
Refused	4 (0.04%)	0 (0.00%)	
Don't Know	5 (0.06%)	1 (0.16%)	
**Marital status, n (%)**			***
Married	4606 (51.65%)	306 (49.51%)	
Widowed	626 (7.02%)	133 (21.52%)	
Divorced	1070 (12.00%)	89 (14.40%)	
Separated	224 (2.51%)	24 (3.88%)	
Never married	1644 (18.43%)	31 (5.02%)	
Living with partner	741 (8.31%)	33 (5.34%)	
Refused	6 (0.07%)	2 (0.32%)	
Don't Know	1 (0.01%)	0 (0.00%)	
**BMI, (kg/m^2^, mean±SD)**	28.65 ± 6.77	30.90 ± 7.72	***
**BMI, n (%)**			***
Normal (< 25)	2830 (32.06%)	121 (20.10%)	
Overweight (25≤BMI<30)	2898 (32.83%)	210 (34.88%)	
Obesity (≥30)	3099 (35.11%)	271 (45.02%)	
**Physical activity, n (%)**			0.183
Yes	5161 (58.07%)	371 (60.82%)	
No	3726 (41.93%)	239 (39.18%)	
**Smoking, n (%)**			***
Non-users	4720 (52.94%)	237 (38.35%)	
Past smoking	1651 (18.52%)	187 (30.26%)	
Current smoking	2545 (28.54%)	194 (31.39%)	
**Hypertension, n (%)**			***
No	6481 (72.77%)	244 (39.48%)	
Yes	2425 (27.23%)	374 (60.52%)	
**Diabetes, n (%)**			***
No	7863 (90.04%)	432 (72.73%)	
Yes	870 (9.96%)	162 (27.27%)	
**Marijuana use**			0.128
Non-users	2108 (42.83%)	66 (37.08%)	
Ever use	2814 (57.17%)	112 (62.92%)	
**Urine creatinine**	125.30 ± 81.05	120.95 ± 76.77	0.195
**Urine albumin**	44.99 ± 345.27	48.35 ± 229.84	0.811

BMI, body mass index; ***P < 0.001.

### The comparation of urine VOCs between RA and non-arthritis subgroups

The comparation results of urine VOCs between the 2 subgroups was summarized in the **Table 2**. The parent compounds corresponding to urine VOCs was summarized in **Table S2**. Participants in the RA subgroup showed higher urine concentrations than the non-arthritis subgroup in terms of AMCC, CEMA, DHBMA, 3HPMA, MHBMA3, PGA and HMPMA. For the other 8 VOCs, no significant difference was detected between the 2 subgroups. In addition, the comparation of VOCs between RA and non-arthritis groups adjusted by sampling weight was summarized in **Table S3**, the analysis results were almost consistent with that adjusted without sampling weight.

**Table 2:** The concentrations of volatile organic compound metabolites (VOCs) in urine of non-arthritis and RA subgroups.

VOCs (urine, ng/ml)	Non-arthritis	RA	*p*
	N=8918, GM (95%CI)	N=618, GM (95%CI)	
2MHA	34.07 (33.19, 34.97)	35.52 (32.18, 39.22)	0.423
3,4-MHA	209.18 (203.51, 215.02)	227.08 (204.86, 251.71)	0.136
AAMA	60.29 (58.95, 61.66)	61.21 (56.35, 66.49)	0.734
**AMCC**	154.40 (150.80, 158.09)	200.06 (183.66, 217.92)	***
ATCA	101.89 (99.50, 104.34)	106.41 (96.81, 116.96)	0.364
SBMA	6.98 (6.83, 7.14)	7.16 (6.53, 7.85)	0.572
CEMA	104.97 (102.69, 107.31)	135.20 (124.47, 146.85)	***
CYMA	5.68 (5.41, 5.97)	6.81 (5.62, 8.24)	0.068
DHBMA	302.48 (297.45, 307.59)	356.32 (335.22, 378.74)	***
2HPMA	36.32 (35.50, 37.17)	39.61 (36.39, 43.11)	0.060
3HPMA	315.23 (307.67, 322.98)	350.48 (319.86, 384.04)	*
MA	148.92 (146.10, 151.79)	156.88 (145.78, 168.82)	0.174
MHBMA3	6.89 (6.70, 7.08)	8.08 (7.28, 8.97)	**
PGA	188.64 (184.59, 192.78)	220.22 (204.49, 237.17)	***
HMPMA	302.30 (294.99 309.80)	365.27 (332.30, 401.51)	***

GM, geometric mean; *p < 0.05; **p < 0.01; ***p < 0.001.

### The comparation of urine VOCs in the RA population with different smoking status

The comparation results of urine VOCs between the 3 subgroups in the RA population was summarized in the **Table S4**. Among the RA population, the current smokers showed significantly higher urine concentrations of 2MHA, 3,4-MHA, AAMA, AMCC, CEMA, CYMA, DHBMA, 3HPMA, MA, MHBMA3, PGA and HMPMA than that of the non-smokers or past smokers. While no significant difference was detected in terms of 2HPMA and SBMA.

### The association between urine VOCs and RA

The quantile logistic regression method was used to analyze the association between VOCs and RA. Firstly, the concentration of each VOCs was divided into 4 quantiles from low to high with equal sample size. The incidence of RA in the quantile 1 (Q1) was set as the reference, and the corresponding odds value (OR) was obtained by comparing the incidence of the other quantiles (Q2-Q4) with that of Q1. The concentration distribution of the 15 VOCs in urine was summarized in the **Table 3**. Then 3 quantile logistic regression models with different covariates were constructed to analyze the association between exposure to VOCs and RA.

**Table 3:** The concentration distribution of various VOCs.

VOCs (urine, ng/ml)	Q1	Q2	Q3	Q4
2MHA	3.54 - 14.1	14.2 - 32.5	32.6 - 86.6	86.7 - 60300
3,4-MHA	5.66 - 82.6	82.7 - 199	200 - 554	555 - 586000
AAMA	1.56 - 29.9	30 - 60	60.1 - 122	123 - 5350
AMCC	4.43 - 75	75.2 - 156	157 - 340	341 - 46300
ATCA	10.6 - 48.7	48.8 - 111	112 - 233	234 - 3250
SBMA	0.354 - 3.49	3.5 - 6.78	6.79 - 13.2	13.3 - 7930
CEMA	4.92 - 54.8	55 - 110	111 - 212	213 - 6480
CYMA	0.354 - 0.919	0.921 - 2.14	2.15 - 63.3	63.4 - 2800
DHBMA	3.71 - 188	189 - 336	337 - 537	538 - 4580
2HPMA	3.75 - 17.9	18 - 35.8	35.9 - 72.7	72.8 - 10100
3HPMA	9.2 - 146	147 - 298	299 - 678	679 - 27400
MA	8.5 - 84.1	84.2 - 153	154 - 269	270 - 27500
MHBMA3	0.424 - 2.86	2.87 - 5.89	5.9 - 16.9	17 - 444
PGA	8.5 - 112	113 - 218	219 - 375	376 - 32200
HMPMA	0.8 - 139	140 - 265	266 - 622	627 - 17000

As shown in **Table 4**, for the analysis between 2MHA and RA, no significant OR was detected in the Q4 and total quantile of the model 1 and model 2. For AAMA, no significant OR was detected in all the quantiles of the non-adjusted model, while a significantly increased OR was found in the Q3 and Q4 of the model 1 and model 2. Meanwhile, a significantly increased OR was detected in the Q3 and Q4 of the non-adjusted and model 1 in terms of AMCC, CEMA and PGA, which was also detected in the Q3 of the model 2. A significantly positive association between RA and CYMA was detected in the model 1. As for DHBMA, a significantly increased OR was detected in the total quantiles of the non-adjusted model, Q3 and Q4 of the model 1, along with the Q4 model 2. And a significantly increased OR of 2HPMA was detected in the Q2 and Q4 of model 1. For MA, a significantly increased OR was detected in the Q4 of all the models. For MHBMA3, a significantly increased OR was detected in the Q3 and Q4 of the model 1. And a significantly positive association between RA and HMPMA was detected in the Q4 of the non-adjusted model and model 1. In addition, it was worth noting that a significant OR was detected over the whole quantiles of the model 1, in terms of 3HPMA. For 3HPMA, the relative OR in the Q2 and Q4 of the model 2 was 1.494 (95%CI: 1.124, 1.985) and 1.621 (95%CI: 1.111, 2.364) respectively. After adding marihuana use as covariates (**Table S5**), the positive association was only detected in the Q4 (OR=2.173, 95%CI:1.021-4.627) of AMCC, and the Q2(OR=2.286, 95%CI: 1.207-4.3307) and Q4 (OR=2.663, 95%CI: 1.288-5.508) of 3HPMA. It was worth noting that the subgroup analysis indicated no significant association between RA and VOCs (**Table S6**). In addition, the non-linear relationship between RA and 15 VOCs was analyzed using the smooth curve fitting method (**Figures S1–15**). The RA incidence increased with the increase of AAMA (**Figure S3**), SBMA (**Figure S6**), DHBMA (**Figure S9**) and MA (**Figure S9**).

**Table 4:** The association between urine VOCs and RA.

VOCs (urine, ng/ml)	Non-adjusted	Model 1	Model 2
	N=9536	N=9533	N=8502
	OR (95% CI)	OR (95% CI)	OR (95% CI)
**2MHA**		Q1 as reference	
**Q2**	0.940 (0.741, 1.192)	1.009 (0.782, 1.304)	0.974 (0.734, 1.291)
**Q3**	1.062 (0.843, 1.339)	1.184 (0.919, 1.527)	1.108 (0.830, 1.481)
**Q4**	1.148 (0.914, 1.442)	1.303 (0.998, 1.702)	1.140 (0.817, 1.592)
**3,4-MHA**		Q1 as reference	
**Q2**	1.130 (0.897, 1.423)	1.023 (0.794, 1.318)	0.863 (0.653, 1.141)
**Q3**	0.940 (0.739, 1.195)	0.966 (0.740, 1.261)	0.807 (0.596, 1.094)
**Q4**	1.182 (0.941, 1.486)	1.154 (0.878, 1.515)	0.828 (0.583, 1.178)
**AAMA**		Q1 as reference	
**Q2**	1.137 (0.900, 1.437)	1.244 (0.963, 1.607)	1.187 (0.895, 1.575)
**Q3**	1.129 (0.893, 1.426)	*1.363 (1.042, 1.784)	*1.366 (1.009, 1.848)
**Q4**	1.134 (0.897, 1.434)	***1.737 (1.291, 2.338)	*1.580 (1.091, 2.289)
**AMCC**		Q1 as reference	
**Q2**	1.165 (0.896, 1.513)	1.112 (0.840, 1.473)	0.955 (0.700, 1.305)
**Q3**	***1.767 (1.387, 2.252)	***1.704 (1.300, 2.234)	*1.451 (1.069, 1.969)
**Q4**	***1.814 (1.425, 2.310)	***1.798 (1.350, 2.396)	1.444 (0.993, 2.101)
**ATCA**		Q1 as reference	
**Q2**	*0.745 (0.590, 0.942)	*0.748 (0.584, 0.959)	0.771 (0.588, 1.009)
**Q3**	*0.774 (0.614, 0.976)	*0.748 (0.580, 0.966)	0.810 (0.615, 1.067)
**Q4**	1.023 (0.823, 1.272)	1.020 (0.787, 1.322)	0.846 (0.634, 1.130)
**SBMA**		Q1 as reference	
**Q2**	1.165 (0.926, 1.466)	1.099 (0.856, 1.410)	1.081 (0.822, 1.420)
**Q3**	1.057 (0.836, 1.337)	0.944 (0.724, 1.230)	0.977 (0.732, 1.305)
**Q4**	1.042 (0.824, 1.318)	0.854 (0.642, 1.136)	0.923 (0.675, 1.262)
**CEMA**		Q1 as reference	
**Q2**	0.989 (0.764, 1.282)	1.027 (0.778, 1.354)	1.026 (0.760, 1.385)
**Q3**	***1.499 (1.183, 1.900)	**1.518 (1.157, 1.991)	*1.361 (1.006, 1.841)
**Q4**	***1.653 (1.308, 2.087)	***1.633 (1.222, 2.180)	1.241 (0.868, 1.774)
**CYMA**		Q1 as reference	
**Q2**	1.152 (0.913, 1.453)	1.197 (0.927, 1.545)	1.118 (0.847, 1.475)
**Q3**	0.947 (0.743, 1.206)	1.231 (0.935, 1.620)	1.107 (0.805, 1.523)
**Q4**	1.249 (0.994, 1.570)	***1.633 (1.246, 2.140)	1.199 (0.769, 1.871)
**DHBMA**		Q1 as reference	
**Q2**	*1.288 (1.001, 1.657)	1.276 (0.971, 1.677)	1.161 (0.862, 1.563)
**Q3**	**1.422 (1.111, 1.821)	*1.459 (1.084, 1.963)	1.308 (0.947, 1.807)
**Q4**	***1.790 (1.411, 2.271)	***1.978 (1.392, 2.810)	*1.601 (1.084, 2.364)
**2HPMA**		Q1 as reference	
**Q2**	1.192 (0.943, 1.506)	**1.419 (1.099, 1.831)	1.253 (0.950, 1.653)
**Q3**	1.065 (0.838, 1.353)	1.307 (0.999, 1.712)	1.097 (0.815, 1.476)
**Q4**	1.236 (0.980, 1.560)	**1.554 (1.174, 2.056)	1.143 (0.825, 1.584)
**3HPMA**		Q1 as reference	
**Q2**	**1.409 (1.114, 1.782)	***1.702 (1.313, 2.207)	**1.494 (1.124, 1.985)
**Q3**	1.114 (0.871, 1.424)	**1.584 (1.195, 2.098)	1.364 (0.992, 1.877)
**Q4**	**1.370 (1.082, 1.735)	***1.999 (1.509, 2.649)	*1.621 (1.111, 2.364)
**MA**		Q1 as reference	
**Q2**	1.109 (0.873, 1.409)	1.212 (0.934, 1.573)	1.251 (0.938, 1.668)
**Q3**	1.104 (0.870, 1.400)	1.302 (0.985, 1.721)	1.322 (0.969, 1.802)
**Q4**	*1.337 (1.063, 1.683)	***1.754 (1.296, 2.375)	*1.532 (1.059, 2.217)
**MHBMA3**		Q1 as reference	
**Q2**	1.190 (0.937, 1.512)	1.134 (0.873, 1.473)	1.050 (0.789, 1.398)
**Q3**	*1.271 (1.004, 1.609)	*1.337 (1.016, 1.760)	1.140 (0.834, 1.557)
**Q4**	1.255 (0.991, 1.590)	*1.427 (1.078, 1.890)	1.027 (0.679, 1.552)
**PGA**		Q1 as reference	
**Q2**	1.260 (0.981, 1.617)	1.271 (0.973, 1.659)	1.125 (0.843, 1.503)
**Q3**	***1.600 (1.259, 2.032)	***1.651 (1.255, 2.171)	*1.358 (1.006, 1.833)
**Q4**	**1.487 (1.167, 1.894)	**1.633 (1.192, 2.238)	1.276 (0.889, 1.831)
**HMPMA**		Q1 as reference	
**Q2**	1.208 (0.949, 1.537)	1.130 (0.868, 1.470)	1.008 (0.757, 1.342)
**Q3**	1.079 (0.842, 1.381)	0.996 (0.746, 1.329)	0.750 (0.541, 1.040)
**Q4**	***1.541 (1.223, 1.940)	**1.538 (1.168, 2.024)	1.231 (0.843, 1.798)

**Non-adjusted:** model without any covariates; **model 1:** model adjusted by age, gender, race, educational level, marital status, urine creatinine and albumin; **model 2:** model adjusted by age, gender, race, educational level, marital status, total energy intake, physical activity, smoking, hypertension, diabetes, urine creatinine and albumin. (*p<0.05; **p<0.01; ***p<0.001).

## Discussion

In this study, we performed a cross-sectional study to systematically investigate the association between the exposure to specific VOC and risk of RA. The VOC concentration in human could be tested in blood, urine, breath and even sweat. However, direct testing of VOC in blood, urine or breath may yield inaccurate results due to the volatilization characteristic of VOC. Although the primary route of human exposure to VOC is through lung inhalation (23), VOC in the body are mainly metabolized through the digestive system, especially the liver digestive enzymes, to form water-soluble VOC metabolites (VOCs) (24). Most VOCs are specific to relative parent compounds, and finally excreted from the body in the urine (25). Therefore, the specificity to parent compounds and stable physicochemical properties makes VOCs more suitable biomarkers of environmental VOC exposures. In this study, we collected the data of VOCs in urine to represent the exposure degree of VOC. As shown in the results part, a total of 9536 participants with 15 VOCs in urine were included from 6 survey cycles of the NHANES program. All the participants were grouped into RA or non-arthritis subgroups, and the baseline data differs significantly between the 2 subgroups. The proportion of smokers in the RA subgroups was significantly higher than that in the non-arthritis subgroups, which was consistent with previous studies demonstrating that smoking was identified as risk factor of RA. And also, we found that smokers with RA have higher VOCs than non-smokers, while whether smoking plays a role in the association between VOCs and RA remained yet clarified. Meanwhile, we also found that the incidence of hypertension and diabetes in the RA subgroups was significantly higher than that in the non-arthritis subgroups. It was found that the frequencies of hypertension, dyslipidemia, and diabetes in the RA population was significantly higher than that in the general population, which was consistent with our findings and proved from the side that RA is a systemic autoimmune disease (26). The VOCs concentration in urine between the 2 subgroups was then compared, with significantly higher concentration of 7 VOCs detected in the RA subgroups than the non-arthritis subgroup {AMCC, CEMA, DHBMA, 3HPMA, MHBMA3, PGA and HMPMA}. The difference of VOCs concentration pattern between the 2 groups suggested that the exposure to some VOC was more serious in the RA subgroup than the non-arthritis group.

In order to further analyze the association between exposure to VOC and RA, 3 quantile regression models with different covariates were constructed. After analysis, 9 VOCs were identified to be positively associated with RA at different concentration quantiles, including CEMA, 3HPMA, AAMA, CYMA, DHBMA, HMPMA, AMCC, PGA and MA. As shown in **Table S2**, relative parent compounds (acrolein, acrylamide, acrylonitrile, 1,3-butadiene, crotonaldehyde, *N,N*-Dimethylformamide, Ethylbenzene, styrene) to the 9 VOCs were summarized according to previous studies. After adjusted by all the covariates, there were 6 VOCs remained to be associated with RA at different concentration quantiles, including CEMA, 3HPMA, DHBMA, AMCC, PGA and MA. Most of these parent compounds have been identified as hazardous environmental pollutants by the United States Environmental Protection and Agency (U.S. EPA).

Among these VOCs, the 3HPMA stand out because a statistically significant OR was detected over the whole quantiles of all the 3 models. Except for 3HPMA, CEMA was another metabolite of acrolein, which was also detected positively associated with RA in this study. Acrolein is ubiquitous in nature and living environments and widely applied as an intermediate to produce acrylic acid, which possesses super-absorbent properties and used as raw materials for diapers, paints and coatings (27, 28). The toxicity of acrolein manifests as irritation of the mucocutaneous membrane, such as eyes, nose and lungs, of which the stimulus only works at the moment of contact (29). However, less evidence has been provided to demonstrate the exposure of acrolein as a cause of diseases (30). Previous studies observed increased protein-conjugated acrolein in plasm at early stage of several diseases, including renal failure (31), Alzheimer’s disease (32), brain infarction (33, 34) and primary Sjogren’s syndrome (35). These studies suggested that the increased protein-conjugated acrolein may be positively associated with the disease progression. In addition, it was worth noting that 3 studies reported the potential association between blood acrolein and RA (36–38). While two studies simply used free acrolein as an indicator of inflammation during RA progression (36, 38). And the other study also observed increased protein-conjugated acrolein in the early stage of RA (38). However, neither free nor protein-conjugated acrolein in the blood can fully represent the body’s exposure level to acrolein. Nevertheless, their observation of increased acrolein in blood sample of RA patients was consistent with our findings, synergistically providing strong evidence the exposure to acrolein may be an important risk factor for RA.

Except for acrolein, there were also some VOC found to associate positively with RA, while the statistical evidence for these is significant in Q3, Q4 or total quantiles, not in the whole quantile. These VOC, with statistically weaker significance, included acrylamide, acrylonitrile, crotonaldehyde, 1,3-Butadiene, *N,N*-Dimethylformamide and Ethylbenzene, styrene. And all these VOC were firstly found to be positively associated with the RA. The acrylamide has been widely used for producing polyacrylamide polymers applied as flocculants, and it was reported that the exposure to acrylamide was associated with the impairment of central and peripheral nervous system (39). In addition, the acrylamide has been early identified as a genotoxic and carcinogenic compound (40). The significant association between acrylamide exposure and RA indicated that the toxic effects of acrylamide are not only neurotoxic, but are likely to be systemic, since rheumatoid arthritis is a systemic autoimmune disease.

Acrylonitrile has been extensively applied as raw materials to manufacture acrylic fibers, plastics, synthetic rubbers, and acrylamide (41). It was pointed out that smoking were important sources of acrylonitrile exposure in the US population (42). The exposure to acrylonitrile at concentrations 20 ppm would result in headaches, nausea and dizziness (43), and acrylonitrile was identified as possibly carcinogenic to humans (Group 2B) (44). However, there were little studies reporting the association between acrylonitrile exposure and specific diseases. In this study, urinary CYMA, as the major metabolite of acrylonitrile, was firstly detected to be associated with RA. However, it was worth noting that the positive association attenuated after adjusting for smoking in Model 2. Similarly, the HMPMA, as the metabolites of crotonaldehyde, was firstly found to be positively associated with RA. The major source of crotonaldehyde is tobacco smoke (45), and it was reported that the crotonaldehyde exposure could result in vascular injury through Wnt and ErbB signaling pathways (46). While little is known about the effect of crotonaldehyde exposure on RA incidence. Considering that smoking is major risk factor for RA and the main source of acrylonitrile and crotonaldehyde exposure, our findings suggested that acrylonitrile and crotonaldehyde may be the main active ingredient in smoking induced RA.

Similarly, 1,3-butadiene, as the parent compound of DHBMA, was mainly produced from tobacco smoke and reported to cause damage on human health (47). The toxicity of 1,3-butadiene manifests during the metabolism process *in vivo*, including enzyme disorders, GSH depletion, and saturable metabolism (48). The 1,3-butadiene has been identified as known carcinogens, with several epidemiological studies demonstrating that the exposure to 1,3-butadiene was positively associated with increased incidence of various cancers, such as colorectal, prostate, lung cancers and hematological system neoplasms (25, 49). In addition, several studies have reported the harmful effect of 1,3-butadiene exposure on cardiovascular diseases, which is absent in terms of RA (50). This is the first to discover the positive association between DHBMA and RA, and these findings suggested that the exposure to 1,3-butadiene is significantly associated with RA.

In addition, the *N,N*-Dimethylformamide, as the parent compound of AMCC, was reported to specifically induce liver impairment. The potential mechanism of targeted harmful effects of *N,N*-Dimethylformamide on liver was mainly contributed to oxidative stress caused by reduced GSH level and increased reactive oxygen species (51). And it was also found that the harmful mechanism of *N,N*-Dimethylformamide involved the activation of NLRP3 inflammasome (52), suggesting the induced inflammatory reaction may also be the underlying cause. The exposure to ethylbenzene, styrene was also reported to cause harmful effect on the central nervous, immune and reproductive systems (25, 53), while its potential harmful cause of RA was firstly found in this study. These findings can help us explain why *N,N*-Dimethylformamide and ethylbenzene, styrene also cause RA, since excessive oxidative stress and inflammatory reaction are also the main mechanism of RA (13).

In conclusion, we identified several VOC was significantly associated with RA, through analyzing the association between the VOCs in urine and RA. These findings would provide us epidemiological evidence about the association between environment pollutants and RA.

### Strength and limitation

It has been reported that VOC, a common environment pollutant, was closely associated with some autoimmune diseases, while whether VOC exposure or which VOC leads to RA is yet clarified. To our best knowledge, this is the first study to systematically investigate the association between VOC exposure and RA. Several VOCs were identified to be associated with RA, giving us novel inspiration for understanding the development of RA and preventing RA in the environmental perspective. This study was performed on the basis of the NHANES program, and the population inclusion was scientifically designed, which could authoritatively represent the health and nutrition status of the USA population. A total of 3 logistic regression models were constructed for analysis and the analysis results still remained stable after analyzing using the 3 models. Nevertheless, some limitations should not be ignored when generalizing the conclusion. Firstly, all the data used in this cross-sectional study was collected at the same time, so it was unable to the infer the casual relationship between VOC exposure and RA. More cohort or prospective studies investigating the association between RA and VOC may be an effective method to explore their causality. Secondly, the precision of the conclusions may be compromised, because some VOCs with more than a quarter of unqualified data was excluded when using quantile regression model. Thirdly, the diagnosis of RA condition was defined through questionnaire survey method, which may lead to misdiagnose or missed diagnosis of some participants and impair the evidence level of our conclusion. In addition, it was difficult to locate the pathogenic site of RA exactly due to the limitation of data availability, which should be considered carefully when generalizing our conclusions. In general, more prospective studies and related experimental studies are needed to further validate the conclusions of this study.

## Conclusion

In this study, we analyzed whether VOCs in urine program associate with RA using the dataset from the NHANES. A total of 9536 participants with 15 VOCs in urine were eligible for final analysis. 3 logistic regression models were constructed for correlation analysis, identifying several VOCs to be significantly associated with RA. The relative parent compounds of these VOCs included acrolein, acrylonitrile and 1,3-butadiene, xylene, acrylamide, crotonaldehyde, *N,N*-Dimethylformamide, ethylbenzene and propylene oxide. These findings suggested that the VOC exposure significantly associated with RA. And also, more prospective studies and related experimental studies are needed to further validate the conclusions of this study.

## Data availability statement

The raw data supporting the conclusions of this article will be made available by the authors, without undue reservation.

## Ethics statement

The investigation protocol of NHANES program was reviewed and approved by the National Center for Health Statistics (NCHS), a division of Centers for Disease Control and Prevention (CDC). Before inclusion, all the participants received an informed consent form setting out details of this program, and would sign their consent form.

## Author contributions

TL finished the writing of the manuscript. TL and JY complete the data analysis. HQ made the tables and figures. YH and JY reviewed and revised the manuscript. YH repeated the data analysis and supervised the whole process. All authors contributed to the article and approved the submitted version.
