# Supplementary material for: The exposure to volatile organic chemicals associates positively with rheumatoid arthritis: a cross-sectional study from the NHANES program
Source: Front Immunol. 2023 Jun 19;14:1098683. doi: 10.3389/fimmu.2023.1098683 (PMC10317299; doi:10.3389/fimmu.2023.1098683)
Supplement: Supplementary file 1 [file DataSheet_1.docx]

**Supplementary materials**

**Table S1. Baseline Data of Included Participants adjusted by sampling weight.**

| **characteristics** | **No arthritis (mean, 95%CI)** | **RA (mean, 95%CI)** | ***p*** |
| --- | --- | --- | --- |
| **Age (years)** | 42.93 (42.36 ,43.50) | 57.80 (56.19 ,59.42) | <0.0001 |
| **Gender, n (%)** |  |  | 0.0084 |
| Male | 52.37 (51.10 ,53.64) | 43.83 (37.80 ,50.04) |  |
| female | 47.63 (46.36 ,48.90) | 56.17 (49.96 ,62.20) |  |
| **Race hispanic origin, n (%)** |  |  | 0.0009 |
| Mexican American | 9.35 (7.91 ,11.01) | 7.15 (4.66 ,10.80) |  |
| Other Hispanic | 6.49 (5.49 ,7.67) | 4.75 (3.27 ,6.87) |  |
| Non-Hispanic White | 62.32 (59.25 ,65.30) | 65.34 (60.01 ,70.31) |  |
| Non-Hispanic Black | 12.68 (11.01 ,14.57) | 17.29 (13.79 ,21.47) |  |
| Other Race | 9.16 (8.14 ,10.29) | 5.47 (3.68 ,8.07) |  |
| **Education level (%)** |  |  | <0.0001 |
| Less Than 9th Grade | 4.66 (4.10 ,5.29) | 8.96 (6.52 ,12.20) |  |
| 9-11th Grade | 10.11 (9.01 ,11.33) | 13.71 (10.68 ,17.43) |  |
| High School Graduate | 23.90 (22.47 ,25.39) | 27.53 (22.47 ,33.24) |  |
| Some College or AA degree | 31.37 (29.84 ,32.94) | 36.31 (30.67 ,42.36) |  |
| College Graduate or above | 29.89 (27.49 ,32.40) | 13.44 (9.45 ,18.77) |  |
| Refused | 0.05 (0.01 ,0.19) | 0.00 (0.00 ,0.00) |  |
| Don't Know | 0.02 (0.01 ,0.05) | 0.05 (0.01 ,0.37) |  |
| **Marital status (%)** |  |  | <0.0001 |
| Married | 53.69 (51.73 ,55.64) | 54.68 (49.17 ,60.07) |  |
| Widowed | 4.88 (4.32 ,5.50) | 16.27 (13.37 ,19.66) |  |
| Divorced | 11.07 (10.22 ,11.98) | 12.78 (9.74 ,16.59) |  |
| Separated | 2.05 (1.67 ,2.52) | 3.38 (2.02 ,5.61) |  |
| Never married | 19.09 (17.36 ,20.93) | 6.30 (3.97 ,9.86) |  |
| Living with partner | 9.17 (8.23 ,10.21) | 6.52 (4.06 ,10.30) |  |
| Refused | 0.06 (0.02 ,0.18) | 0.07 (0.02 ,0.33) |  |
| Don't Know | 0.00 (0.00 ,0.02) | 0.00 (0.00 ,0.00) |  |
| **BMI, (kg/m^2^, mean±SD)** | 28.64 (28.41 ,28.87) | 29.92 (29.26 ,30.58) | 0.0003 |
| **BMI, n (%)** |  |  | 0.0337 |
| Normal (< 25) | 32.31 (30.67 ,33.99) | 25.38 (20.42 ,31.06) |  |
| Overweight (25≤BMI<30) | 32.24 (30.69 ,33.82) | 33.38 (28.47 ,38.66) |  |
| Obesity (≥30) | 35.45 (33.89 ,37.05) | 41.25 (36.29 ,46.39) |  |
| **Physical activity, n (%)** |  |  | 0.6189 |
| Yes | 53.28 (51.28 ,55.27) | 54.99 (48.74 ,61.09) |  |
| No | 46.72 (44.73 ,48.72) | 45.01 (38.91 ,51.26) |  |
| **Smoking, n (%)** |  |  | <0.0001 |
| No | 51.62 (49.73 ,53.50) | 36.41 (30.81 ,42.42) |  |
| Ever smoking | 20.14 (18.82 ,21.52) | 26.20 (21.56 ,31.43) |  |
| Current smoking | 28.25 (26.72 ,29.82) | 37.39 (31.94 ,43.17) |  |
| **Hypertension, n (%)** |  |  | <0.0001 |
| No | 75.21 (73.74 ,76.62) | 50.19 (44.76 ,55.62) |  |
| Yes | 24.79 (23.38 ,26.26) | 49.81 (44.38 ,55.24) |  |
| **Diabetes, n (%)** |  |  | <0.0001 |
| No | 92.57 (91.94 ,93.15) | 79.38 (74.40 ,83.59) |  |
| Yes | 7.43 (6.85 ,8.06) | 20.62 (16.41 ,25.60) |  |
| **Marijuana use** |  |  | 0.7462 |
| Never | 36.86 (34.56 ,39.22) | 35.44 (27.71 ,44.01) |  |
| Ever | 63.14 (60.78 ,65.44) | 64.56 (55.99 ,72.29) |  |
| **Urine creatinine** | 119.60 (117.22 ,121.97) | 112.50 (105.85 ,119.15) | 0.0439 |
| **Urine albumin** | 30.58 (26.30 ,34.86) | 44.51 (5.67 ,83.35) | 0.4874 |

**Table S2. Relative parent compounds to VOCs detected in this study.**

| **Parent compounds (VOC)** | **VOCs detected in urine (abbreviation)** |
| --- | --- |
| Acrolein | N-Acetyl-S-(2-carboxyethyl)-L-cysteine (CEMA) |
|  | N-Acetyl-S-(3-hydroxypropyl)-L-cysteine (3HPMA) |
| Acrylamide | N-Acetyl-S-(2-carbamoylethyl)-L-cysteine (AAMA) |
| Acrylonitrile | N-Acetyl-S-(2-cyanoethyl)-L-cysteine (CYMA) |
| 1,3-Butadiene | N-Acetyl-S-(3,4-dihydroxybutyl)-L-cysteine (DHBMA) |
|  | N-Acetyl-S-(4-hydroxy-2-butenyl)-L-cysteine (MHBMA3) |
| Crotonaldehyde | N-Acetyl-S-(3-hydroxypropyl-1-methyl)-L-cysteine (HMPMA) |
| Cyanide | 2-Aminothiazoline-4-carboxylic acid (ATCA) |
| *N,N*-Dimethylformamide | N-Acetyl-S-(N-methylcarbamoyl)-L-cysteine (AMCC) |
| Ethylbenzene, styrene | Phenylglyoxylic acid (PGA) |
|  | Mandelic acid (MA) |
| Propylene oxide | N-Acetyl-S-(2-hydroxypropyl)-L-cysteine (2HPMA) |
| Toluene | N-Acetyl-S-(benzyl)-L-cysteine (SBMA) |
| Xylene | 2-Methylhippuric acid (2MHA) |
|  | 3- and 4-Methylhippuric acid (3,4-MHA) |

The table was made with reference to this study: Li, A.J., V.K. Pal, and K. Kannan, *A review of environmental occurrence, toxicity, biotransformation and biomonitoring of volatile organic compounds.* Environmental Chemistry and Ecotoxicology, 2021. **3**: p. 91-116

**Table S3.** The concentrations of volatile organic compound metabolites (VOCs) in urine of non-arthritis and RA subgroups adjusted by sampling weight.

| **VOCs (urine, ng/ml)** | **Non-arthritis (mean, 95% CI)** | **RA (mean, 95% CI)** | ***p*** |
| --- | --- | --- | --- |
| 2MHA | 82.68 (72.02 ,93.33) | 72.64 (62.47 ,82.82) | 0.1636 |
| 3,4-MHA | 562.34 (501.00 ,623.68) | 522.87 (449.86 ,595.88) | 0.3840 |
| AAMA | 104.42 (99.09 ,109.75) | 104.62 (91.03 ,118.20) | 0.9788 |
| AMCC | 301.57 (285.16 ,317.98) | 378.52 (327.65 ,429.39) | 0.0036 |
| ATCA | 166.07 (159.43 ,172.70) | 181.92 (156.93 ,206.90) | 0.2107 |
| SBMA | 12.70 (11.90 ,13.49) | 55.55 (-25.80 ,136.90) | 0.3045 |
| CEMA | 167.99 (160.28 ,175.69) | 221.91 (196.55 ,247.27) | 0.0001 |
| CYMA | 66.29 (60.76 ,71.83) | 79.78 (64.58 ,94.98) | 0.0952 |
| DHBMA | 391.12 (378.86 ,403.38) | 437.65 (408.32 ,466.98) | 0.0039 |
| 2HPMA | 75.63 (69.35 ,81.92) | 95.20 (44.07 ,146.34) | 0.4582 |
| 3HPMA | 615.94 (575.11 ,656.77) | 739.07 (624.65 ,853.49) | 0.0501 |
| MA | 224.96 (213.81 ,236.12) | 230.96 (206.13 ,255.80) | 0.6552 |
| MHBMA3 | 16.71 (15.56 ,17.85) | 21.28 (17.72 ,24.84) | 0.0160 |
| PGA | 294.05 (278.52 ,309.58) | 306.81 (269.09 ,344.53) | 0.5539 |
| HMPMA | 627.10 (588.42 ,665.77) | 798.77 (666.31 ,931.23) | 0.0158 |

**Table S4.** The concentrations of urine volatile organic compound metabolites (VOCs) of RA population grouped by different smoking status.

| **Smoking status** | **Never** | **Ever** | **Current** | ***p*** |
| --- | --- | --- | --- | --- |
| **VOCs (urine, ng/ml)** | N=237, Mean(95%CI) | N=187, Mean(95%CI) | N=194, Mean(95%CI) |  |
| 2MHA | 40.00 ± 67.95 | 52.86 ± 82.35 | 129.28 ± 93.24 | <0.001 |
| 3,4-MHA | 265.57 ± 489.84 | 294.87 ± 520.62 | 980.01 ± 808.57 | <0.001 |
| AAMA | 62.00 ± 65.06 | 76.75 ± 94.12 | 181.79 ± 201.06 | <0.001 |
| AMCC | 190.68 ± 232.93 | 209.44 ± 185.27 | 652.37 ± 522.63 | <0.001 |
| ATCA | 195.41 ± 226.67 | 165.40 ± 185.65 | 236.01 ± 265.27 | 0.010 |
| SBMA | 13.32 ± 29.58 | 59.27 ± 580.17 | 19.45 ± 60.20 | 0.303 |
| CEMA | 125.23 ± 104.25 | 158.71 ± 167.53 | 405.72 ± 347.42 | <0.001 |
| CYMA | 10.18 ± 46.68 | 14.15 ± 73.54 | 214.19 ± 198.51 | <0.001 |
| DHBMA | 395.87 ± 262.97 | 458.00 ± 323.36 | 536.75 ± 358.93 | <0.001 |
| 2HPMA | 98.89 ± 469.67 | 53.84 ± 109.28 | 97.13 ± 102.32 | 0.248 |
| 3HPMA | 284.61 ± 283.91 | 327.19 ± 351.20 | 1553.83 ± 1396.04 | <0.001 |
| MA | 167.15 ± 125.56 | 196.85 ± 391.74 | 355.59 ± 286.34 | <0.001 |
| MHBMA3 | 6.19 ± 7.04 | 6.82 ± 9.13 | 47.12 ± 37.68 | <0.001 |
| PGA | 250.86 ± 185.76 | 268.00 ± 210.69 | 429.88 ± 373.18 | <0.001 |
| HMPMA | 290.99 ± 300.79 | 322.02 ± 377.66 | 1754.48 ± 1468.87 | <0.001 |

*: ***p* < 0.05;** **: ***p* < 0.01;** ***: ***p* < 0.001.**

**Table S5. T**he association analysis between urine VOCs and RA.

| **VOCs (urine, ng/ml)** | **Model 3** | **VOCs (urine, ng/ml)** | **Model 3** |
| --- | --- | --- | --- |
|  | N=4803 |  | N=4803 |
|  | OR (95% CI) *P* |  | OR (95% CI) *P* |
| **2MHA** |  | **DHBMA** |  |
| **Q2** | 0.662 (0.365, 1.201) 0.17481 | **Q2** | 1.255 (0.730, 2.157) 0.41074 |
| **Q3** | 0.877 (0.493, 1.559) 0.65512 | **Q3** | 1.427 (0.807, 2.524) 0.22205 |
| **Q4** | 0.855 (0.454, 1.609) 0.62630 | **Q4** | 1.596 (0.825, 3.086) 0.16477 |
| **3,4-MHA** |  | **2HPMA** |  |
| **Q2** | 0.939 (0.515, 1.715) 0.83841 | **Q2** | 1.073 (0.617, 1.866) 0.80290 |
| **Q3** | 0.845 (0.456, 1.566) 0.59199 | **Q3** | 1.275 (0.739, 2.197) 0.38261 |
| **Q4** | 0.838 (0.421, 1.667) 0.61364 | **Q4** | 0.853 (0.462, 1.575) 0.61169 |
| **AAMA** |  | **3HPMA** |  |
| **Q2** | 1.283 (0.695, 2.369) 0.42481 | **Q2** | 2.286 (1.207, 4.330) 0.01118 |
| **Q3** | 1.757 (0.960, 3.215) 0.06757 | **Q3** | 1.909 (0.973, 3.743) 0.05992 |
| **Q4** | 1.980 (0.994, 3.944) 0.05194 | **Q4** | 2.663 (1.288, 5.508) 0.00823 |
| **AMCC** |  | **MA** |  |
| **Q2** | 1.340 (0.675, 2.657) 0.40262 | **Q2** | 1.203 (0.672, 2.155) 0.53447 |
| **Q3** | 1.868 (0.960, 3.635) 0.06588 | **Q3** | 1.330 (0.738, 2.397) 0.34271 |
| **Q4** | 2.173 (1.021, 4.627) 0.04407 | **Q4** | 1.510 (0.779, 2.926) 0.22221 |
| **ATCA** |  | **MHBMA3** |  |
| **Q2** | 0.481 (0.282, 0.820) 0.00714 | **Q2** | 1.486 (0.787, 2.805) 0.22143 |
| **Q3** | 0.627 (0.382, 1.031) 0.06570 | **Q3** | 1.613 (0.846, 3.075) 0.14676 |
| **Q4** | 0.656 (0.398, 1.082) 0.09903 | **Q4** | 1.411 (0.660, 3.014) 0.37446 |
| **SBMA** |  | **PGA** |  |
| **Q2** | 1.119 (0.672, 1.864) 0.66593 | **Q2** | 1.087 (0.626, 1.887) 0.76608 |
| **Q3** | 1.055 (0.621, 1.793) 0.84227 | **Q3** | 1.339 (0.781, 2.297) 0.28836 |
| **Q4** | 1.095 (0.622, 1.925) 0.75382 | **Q4** | 1.116 (0.600, 2.079) 0.72844 |
| **CEMA** |  | **HMPMA** |  |
| **Q2** | 1.313 (0.706, 2.443) 0.38920 | **Q2** | 1.556 (0.856, 2.827) 0.14702 |
| **Q3** | 1.818 (0.992, 3.332) 0.05307 | **Q3** | 0.945 (0.476, 1.877) 0.87112 |
| **Q4** | 1.610 (0.813, 3.189) 0.17222 | **Q4** | 1.742 (0.850, 3.568) 0.12924 |
| **CYMA** |  |  |  |
| **Q2** | 1.137 (0.600, 2.154) 0.69418 |  |  |
| **Q3** | 1.565 (0.820, 2.988) 0.17429 |  |  |
| **Q4** | 1.781 (0.789, 4.021) 0.16446 |  |  |

**Model 3:** model adjusted by age, gender, race, educational level, marital status, total energy intake, physical activity, smoking, hypertension, diabetes, urine creatinine, albumin and marijuana use.

**Table S6.** the association between urine VOCs and RA in population identified with both non-users smoking and non-users marijuana status.

| **VOCs (urine, ng/ml)** | **Non-adjusted** | **Adjusted** |
| --- | --- | --- |
|  | N= 1634 | N= 1525 |
|  | OR (95% CI) *P* | OR (95% CI) *P* |
| **2MHA** | Q1 as reference | Q1 as reference |
| **Q2** | 0.902 (0.379, 2.148) 0.81625 | 0.634 (0.240, 1.677) 0.35872 |
| **Q3** | 0.902 (0.379, 2.148) 0.81625 | 0.730 (0.266, 2.003) 0.54179 |
| **Q4** | 0.627 (0.241, 1.633) 0.33915 | 0.563 (0.188, 1.691) 0.30622 |
| **3,4-MHA** | Q1 as reference | Q1 as reference |
| **Q2** | 0.816 (0.335, 1.991) 0.65521 | 0.610 (0.217, 1.717) 0.34924 |
| **Q3** | 0.911 (0.383, 2.170) 0.83395 | 0.631 (0.221, 1.807) 0.39125 |
| **Q4** | 0.720 (0.287, 1.809) 0.48464 | 0.658 (0.213, 2.027) 0.46570 |
| **AAMA** | Q1 as reference | Q1 as reference |
| **Q2** | 1.723 (0.671, 4.421) 0.25784 | 1.673 (0.565, 4.957) 0.35299 |
| **Q3** | 1.436 (0.541, 3.809) 0.46750 | 1.455 (0.439, 4.817) 0.53954 |
| **Q4** | 1.286 (0.474, 3.486) 0.62138 | 1.898 (0.509, 7.078) 0.33999 |
| **AMCC** | Q1 as reference | Q1 as reference |
| **Q2** | 2.020 (0.684, 5.963) 0.20295 | 1.556 (0.482, 5.020) 0.45984 |
| **Q3** | 2.030 (0.688, 5.993) 0.19975 | 1.400 (0.415, 4.720) 0.58703 |
| **Q4** | 2.639 (0.932, 7.472) 0.06758 | 1.594 (0.417, 6.096) 0.49556 |
| **ATCA** | Q1 as reference | Q1 as reference |
| **Q2** | 1.178 (0.393, 3.537) 0.76978 | 1.157 (0.334, 4.012) 0.81788 |
| **Q3** | 2.030 (0.755, 5.462) 0.16081 | 2.272 (0.709, 7.276) 0.16700 |
| **Q4** | 2.199 (0.828, 5.844) 0.11391 | 2.630 (0.778, 8.892) 0.11982 |
| **SBMA** | Q1 as reference | Q1 as reference |
| **Q2** | 2.035 (0.756, 5.476) 0.15933 | 1.574 (0.524, 4.731) 0.41872 |
| **Q3** | 2.393 (0.910, 6.289) 0.07683 | 2.091 (0.689, 6.342) 0.19259 |
| **Q4** | 0.998 (0.319, 3.119) 0.99660 | 0.498 (0.120, 2.069) 0.33714 |
| **CEMA** | Q1 as reference | Q1 as reference |
| **Q2** | 1.866 (0.683, 5.094) 0.22358 | 1.713 (0.569, 5.154) 0.33828 |
| **Q3** | 1.870 (0.685, 5.107) 0.22171 | 1.727 (0.544, 5.479) 0.35351 |
| **Q4** | 1.671 (0.602, 4.641) 0.32468 | 1.154 (0.309, 4.308) 0.83130 |
| **CYMA** | Q1 as reference | Q1 as reference |
| **Q2** | 1.299 (0.479, 3.521) 0.60751 | 1.733 (0.567, 5.298) 0.33457 |
| **Q3** | 1.862 (0.735, 4.715) 0.18983 | 2.571 (0.828, 7.981) 0.10218 |
| **Q4** | 1.283 (0.473, 3.477) 0.62484 | 1.214 (0.337, 4.379) 0.76653 |
| **DHBMA** | Q1 as reference | Q1 as reference |
| **Q2** | 1.000 (0.393, 2.545) 1.00000 | 1.011 (0.352, 2.899) 0.98410 |
| **Q3** | 0.887 (0.339, 2.321) 0.80648 | 0.959 (0.281, 3.272) 0.94617 |
| **Q4** | 1.337 (0.557, 3.208) 0.51584 | 1.241 (0.310, 4.973) 0.76034 |
| **2HPMA** | Q1 as reference | Q1 as reference |
| **Q2** | 1.870 (0.685, 5.107) 0.22171 | 1.994 (0.660, 6.020) 0.22085 |
| **Q3** | 1.679 (0.605, 4.664) 0.32001 | 2.150 (0.674, 6.855) 0.19573 |
| **Q4** | 1.856 (0.680, 5.068) 0.22733 | 1.876 (0.559, 6.294) 0.30843 |
| **3HPMA** | Q1 as reference | Q1 as reference |
| **Q2** | 1.126 (0.404, 3.134) 0.82047 | 1.134 (0.377, 3.406) 0.82287 |
| **Q3** | 2.309 (0.940, 5.673) 0.06809 | 2.322 (0.780, 6.911) 0.13015 |
| **Q4** | 0.985 (0.342, 2.834) 0.97780 | 1.157 (0.321, 4.168) 0.82392 |
| **MA** | Q1 as reference | Q1 as reference |
| **Q2** | 1.003 (0.413, 2.435) 0.99558 | 1.161 (0.431, 3.129) 0.76821 |
| **Q3** | 0.605 (0.218, 1.679) 0.33428 | 0.567 (0.166, 1.939) 0.36538 |
| **Q4** | 1.188 (0.508, 2.781) 0.69126 | 1.126 (0.314, 4.044) 0.85555 |
| **MHBMA3** | Q1 as reference | Q1 as reference |
| **Q2** | 0.997 (0.392, 2.539) 0.99579 | 0.764 (0.268, 2.181) 0.61549 |
| **Q3** | 1.323 (0.551, 3.176) 0.53047 | 1.127 (0.383, 3.314) 0.82846 |
| **Q4** | 0.880 (0.336, 2.304) 0.79464 | 0.589 (0.161, 2.149) 0.42259 |
| **PGA** | Q1 as reference | Q1 as reference |
| **Q2** | 2.653 (0.937, 7.510) 0.06617 | 2.090 (0.684, 6.382) 0.19553 |
| **Q3** | 2.449 (0.855, 7.015) 0.09538 | 1.988 (0.586, 6.752) 0.27047 |
| **Q4** | 1.600 (0.519, 4.933) 0.41323 | 1.037 (0.226, 4.748) 0.96305 |
| **HMPMA** | Q1 as reference | Q1 as reference |
| **Q2** | 0.898 (0.343, 2.351) 0.82634 | 0.608 (0.201, 1.833) 0.37648 |
| **Q3** | 1.340 (0.558, 3.216) 0.51222 | 1.208 (0.392, 3.727) 0.74215 |
| **Q4** | 0.995 (0.391, 2.533) 0.99165 | 0.730 (0.191, 2.787) 0.64511 |

**Non-adjusted:** model adjusted without any covariates; **adjusted:** model adjusted by age, gender, race, educational level, marital status, total energy intake, physical activity, hypertension, diabetes, urine creatinine and albumin.


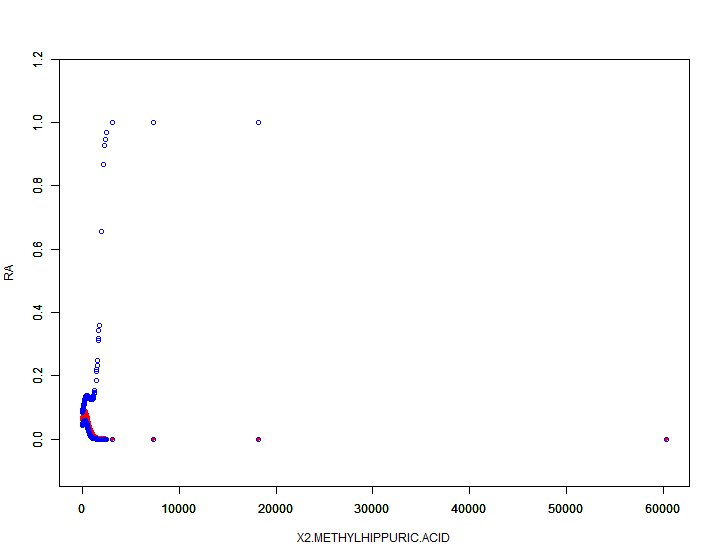


Figure S1. The smooth curve fitting between RA and 2MHA.


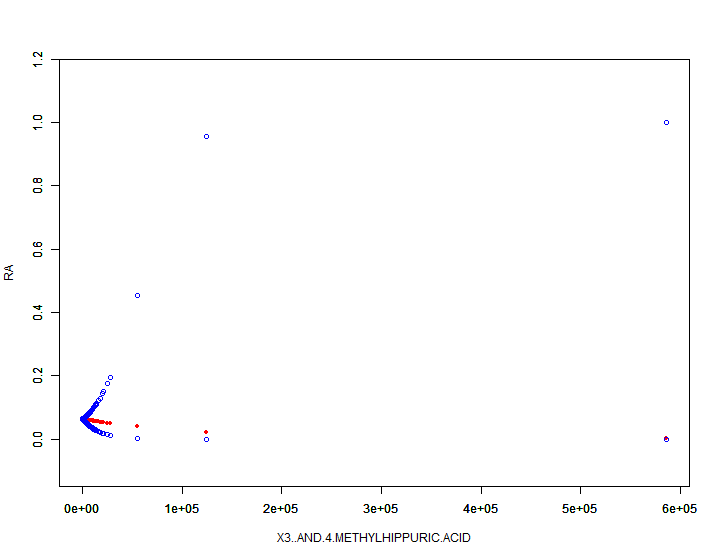


Figure S2. The smooth curve fitting between RA and 3,4-MHA


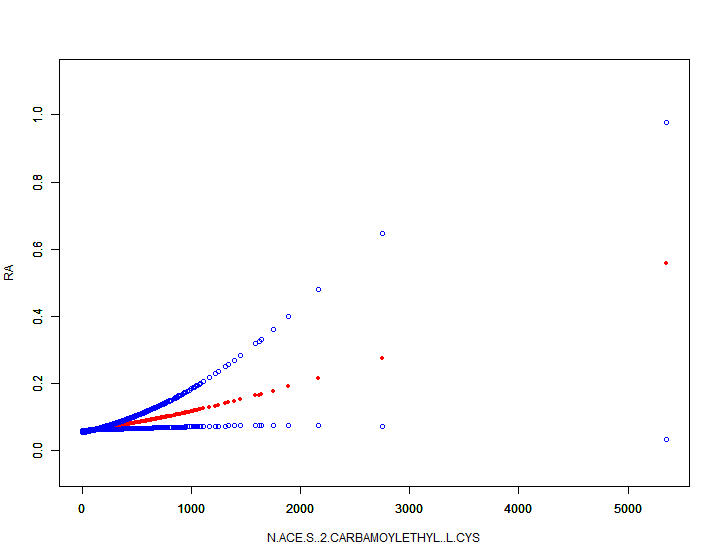


Figure S3. The smooth curve fitting between RA and AAMA


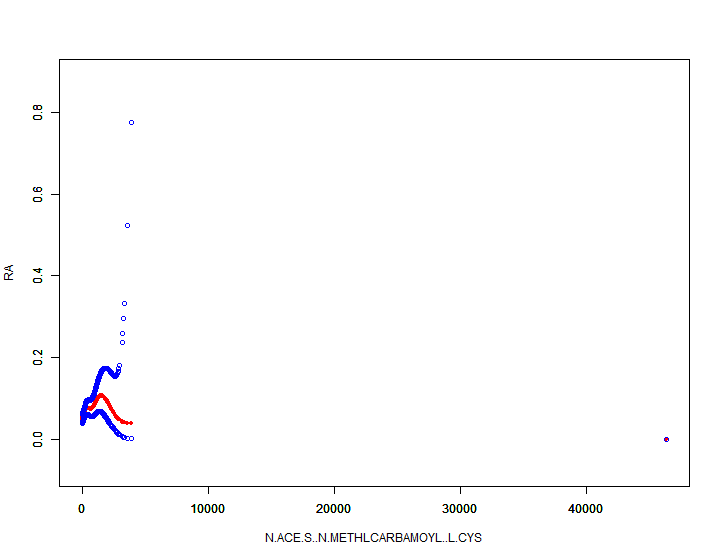


Figure S4. The smooth curve fitting between RA and AMCC


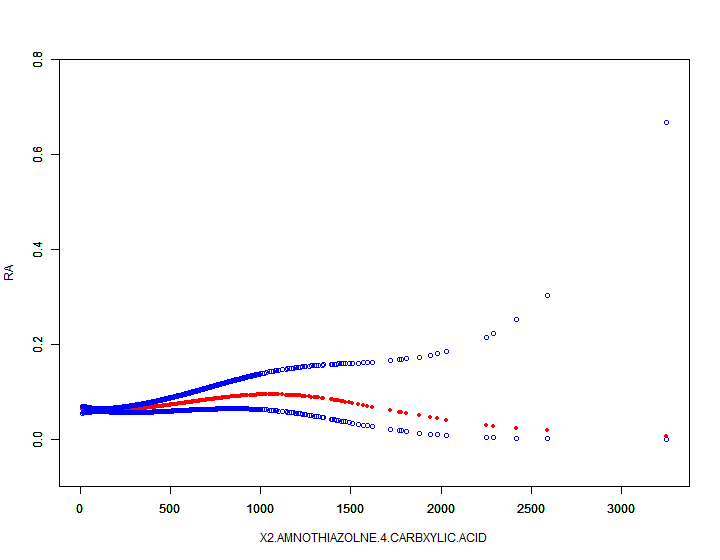


Figure S5. The smooth curve fitting between RA and ATCA


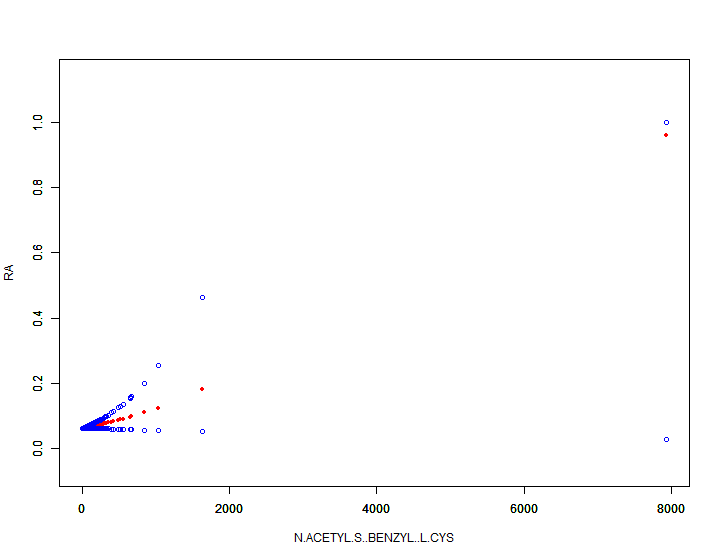


Figure S6. The smooth curve fitting between RA and SBMA


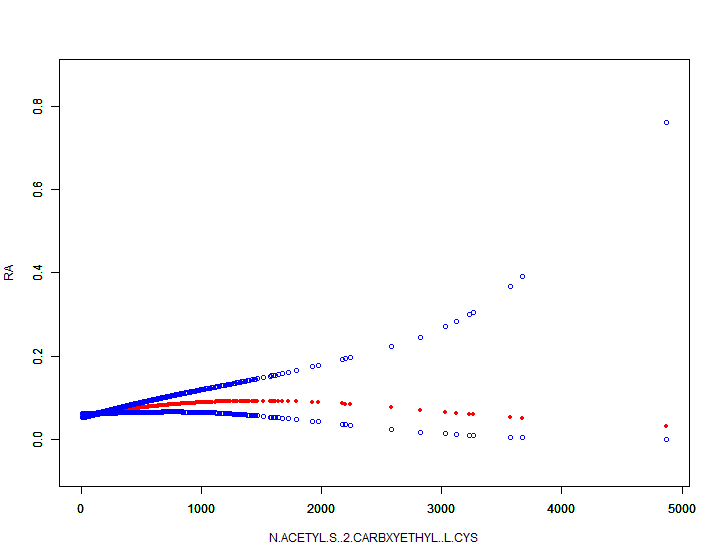


Figure S7. The smooth curve fitting between RA and CEMA


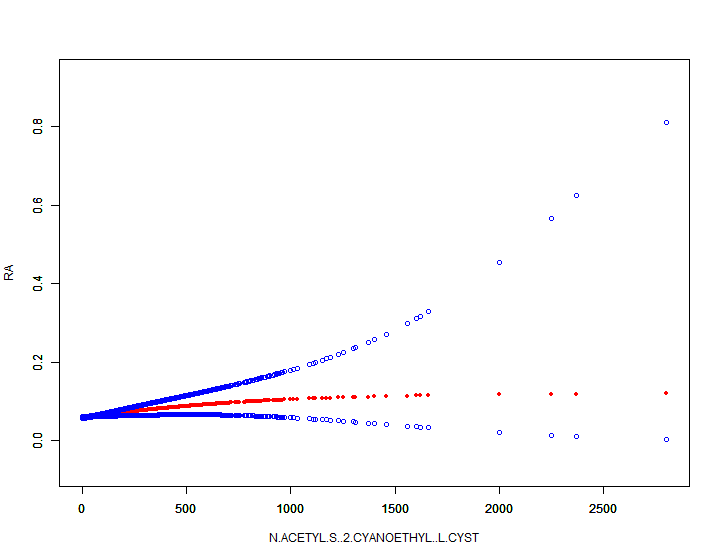


Figure S8. The smooth curve fitting between RA and CYMA


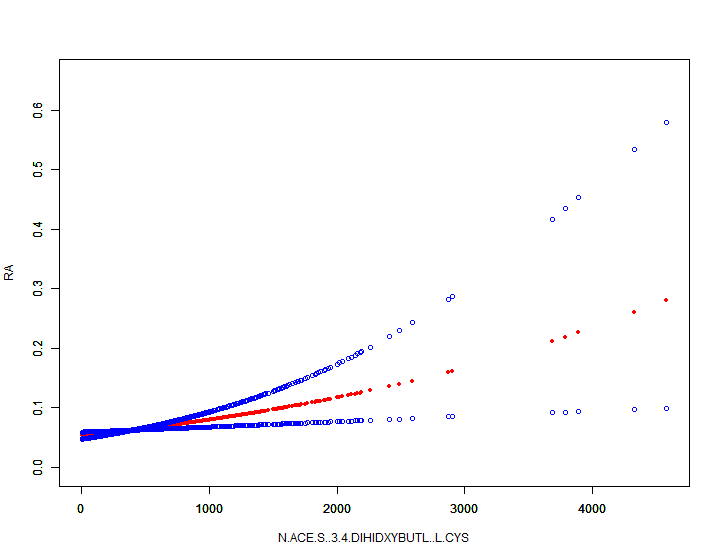


Figure S9. The smooth curve fitting between RA and DHBMA


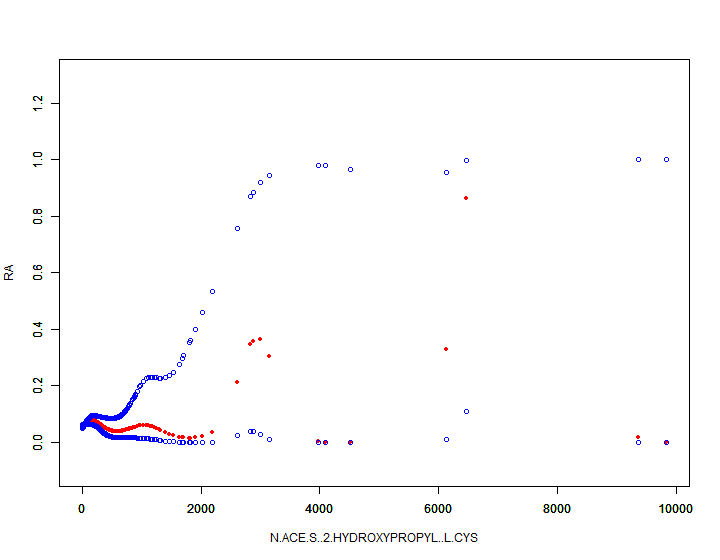


Figure S10. The smooth curve fitting between RA and 2HPMA


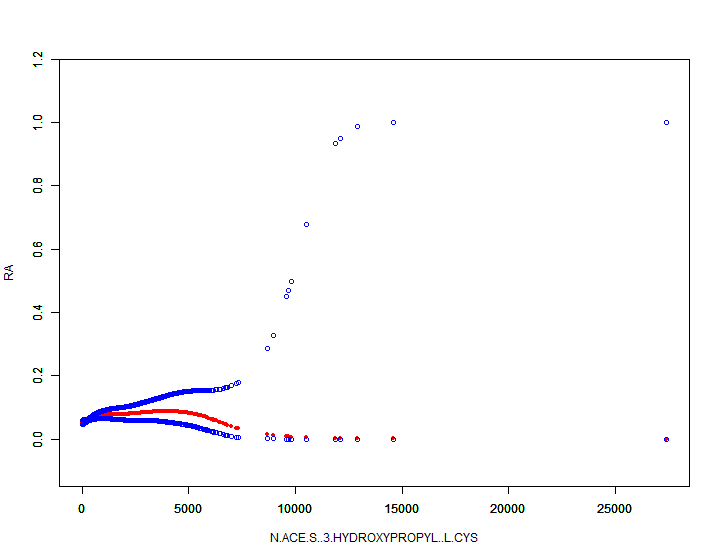


Figure S11. The smooth curve fitting between RA and 3HPMA


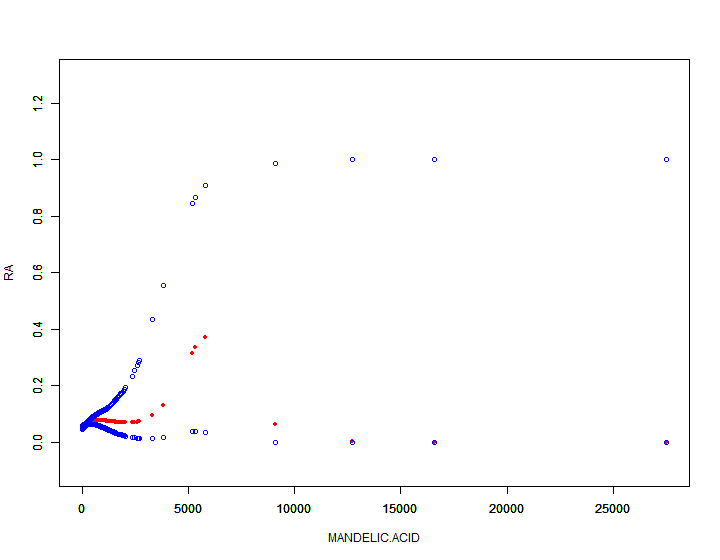


Figure S12. The smooth curve fitting between RA and MA


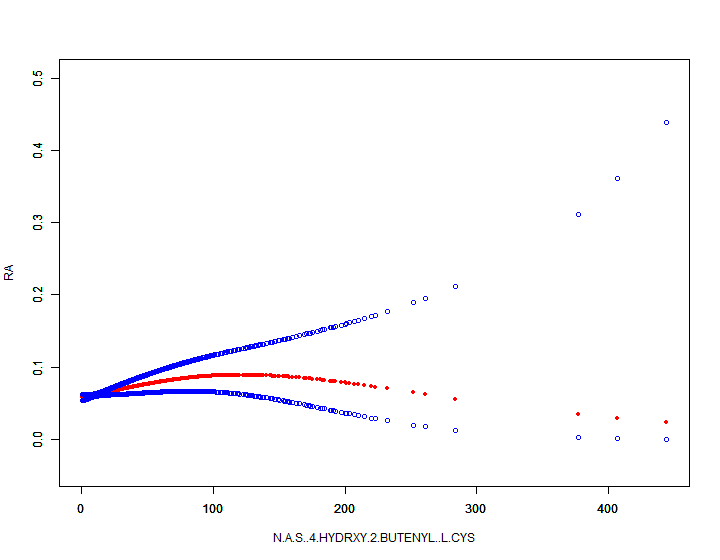


Figure S13. The smooth curve fitting between RA and MHBMA3


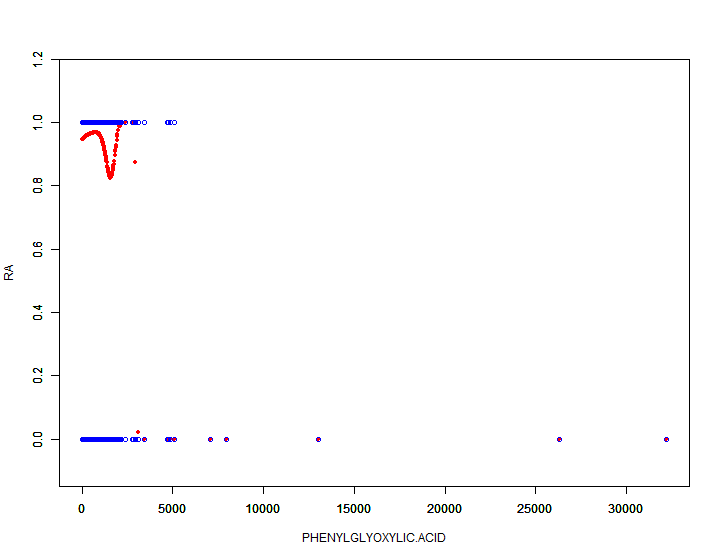


Figure S14. The smooth curve fitting between RA and PGA


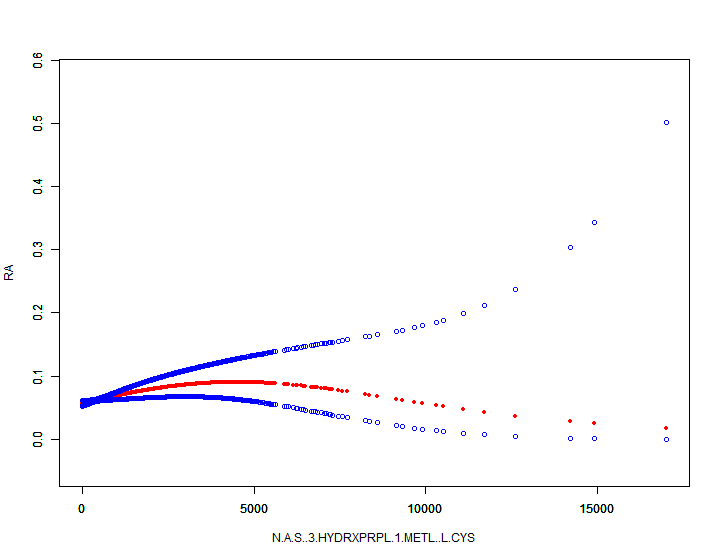


Figure S15. The smooth curve fitting between RA and HMPMA
